# COVID-19 pneumonia: ARDS or not?

**DOI:** 10.1186/s13054-020-02880-z

**Published:** 2020-04-16

**Authors:** Luciano Gattinoni, Davide Chiumello, Sandra Rossi

**Affiliations:** 1 Department of Anesthesiology and Intensive Care Medicine, Medical University of Göttingen, Georg-August-University of Goettingen, Robert-Koch Straße 40, 37075 Göttingen, Germany; 2 SC Anestesia e Rianimazione, Ospedale San Paolo – Polo Universitario, ASST Santi Paolo e Carlo, Dipartimento di Scienze della Salute, Università degli Studi di Milano, Milan, Italy; 3 Azienda Ospedaliero-Universitaria di Parma, Parma, Italy

**Keywords:** COVID-19, ARDS, Mechanical ventilation

Even though it can meet the ARDS Berlin definition [1, 2], the COVID-19 pneumonia is a specific disease with peculiar phenotypes. Its main characteristic is the dissociation between the severity of the hypoxemia and the maintenance of relatively good respiratory mechanics. Indeed, the median respiratory system compliance is usually around 50 ml/cmH_2_O. Of note, the patients with respiratory compliance lower or higher than the median value experience hypoxemia of similar severity. We propose the presence of two types of patients (“non-ARDS,” type 1, and ARDS, type 2) with different pathophysiology. When presenting at the hospital, type 1 and type 2 patients are clearly distinguishable by CT scan (Fig. 1). If the CT scan is not available, the respiratory system compliance and possibly the response to PEEP are the only imperfect surrogates we may suggest.

**Fig. 1:**
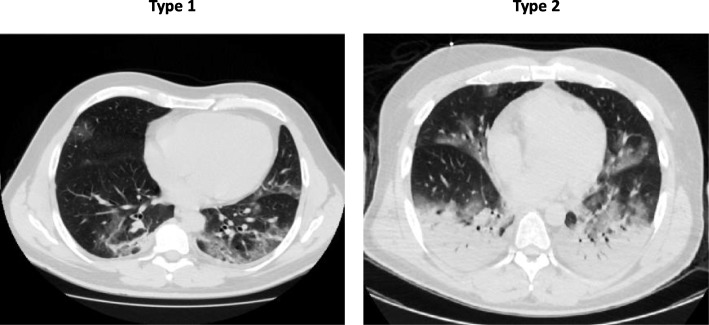
In these 2 patients were recorded the following variables: type 1 lung weight (1192 g), gas volume (2774 ml), percentage of non-aerated tissue (8.4%), venous admixture (56%), P/F (68), and respiratory system compliance (80 ml/cmH_2_O); type 2 lung weight (1441 g), gas volume (1640 ml), percentage of non-aerated tissue (39%), venous admixture (49%), P/F (61), and respiratory system compliance (43 ml/cmH_2_O)

## Type 1: Near normal pulmonary compliance with isolated viral pneumonia

In these patients, severe hypoxemia is associated with respiratory system compliance > 50 ml/cmH_2_O. The lung’s gas volume is high, the recruitability is minimal, and the hypoxemia is likely due to the loss of hypoxic pulmonary vasoconstriction and impaired regulation of pulmonary blood flow. Therefore, severe hypoxemia is primarily due to ventilation/perfusion (V_A_/Q) mismatch. High PEEP and prone positioning do not improve oxygenation through recruitment of collapsed areas, but redistribute pulmonary perfusion, improving the V_A_/Q relationship. Lung CT scans in those patients confirm that there are no significant areas to recruit, but the right-to-left venous admixture is typically around 50%.

## Type 2: Decreased pulmonary compliance

In 20–30% of these COVID-19 patients admitted to the intensive care unit (ICU), severe hypoxemia is associated with compliance values < 40 ml/cmH_2_O, indicating severe ARDS [3]. It is certainly possible that their lower compliance (i.e., lower gas volume and increased recruitability) is due to the natural evolution of the disease, but we cannot exclude the possibility that this severity of damage (increased edema) results in part from the initial respiratory management. Indeed, some of these hypoxemic patients receive CPAP or non-invasive ventilation before ICU admission and present with very high respiratory drives, vigorous inspiratory efforts, and highly negative intrathoracic pressures. Therefore, in addition to viral pneumonia, those patients likely have self-inflicted ventilator-induced lung injury [4].

## Clinical implications

### Before ICU, in non-intubated patients

CPAP and NIV are the first-line treatment when an overwhelming number of patients come to a hospital. These interventions, often applied outside the ICU in emergency rooms or in other medicine wards, usually improve blood oxygenation. A key aspect of care, however, should be the assessment of respiratory drive and the inspiratory efforts. The ideal indicator would be the measurement of the esophageal pressure swings. If impossible, the clinical signs of inspiratory efforts should be carefully scrutinized. If respiratory distress is present, endotracheal intubation should be strongly considered to avoid/limit the transition from type 1 to type 2 by self-induced lung injury.

### In ICU, intubated patients

#### Tidal volume

In type 2 patients, a lower tidal volume should be applied. However, type 1 patients lack the low compliance/high driving pressure prerequisites of ventilator-induced lung injury, even if treated with volumes higher than 6 ml/kg delivered at respiratory rates of 15–20 breaths/min [5]. More liberal tidal volume (7–8 ml/kg) often attenuates dyspnea and may avoid hypoventilation with possible reabsorption atelectasis and hypercapnia.

#### PEEP

The type 1 patients lack the prerequisite for higher PEEP to work (recruitability). PEEP levels should be limited at 8–10 cmH_2_O, since higher levels will decrease pulmonary compliance and can impact right heart function. The type 2 patients are characterized by a reduction of total gas volume and an increase in lung weight and edema. These features may be due to the natural progression of the disease, to bacterial superinfection and/or to self-induced lung injury during the period preceding the intubation. In these patients, a cautious gradual increase of PEEP up to 14–15 cmH_2_O may be beneficial. A decrease in SvO2 during this phase suggests an inadequate cardiac output so that higher PEEP levels for lung recruitment may no longer be useful. Cardiac ultrasound may also be useful for assessing right heart function when increasing PEEP levels.

#### Shunt determination

Calculating the shunt fraction is the best tool to assess oxygenation.

The etCO_2_/PaCO_2_ relationship is a useful tool to quantify efficiency of pulmonary exchange. A ratio < 1 suggests elevated shunt and dead space (areas of lung ventilated and not perfused).

##### Prone positioning

For type 2 patients, prone position could be used as a long-term treatment—as in any form of severe ARDS [6, 7]. However, in type 1 patients, prone positioning should be considered more as a *rescue* maneuver to facilitate the redistribution of pulmonary blood flow, rather than for opening collapsed areas. Long-term prone positioning/supine cycles is of very little benefit in patients with high lung compliance, and it leads to high levels of stress and fatigue in the personnel.

##### Nitric oxide

The oxygenation response to NO is variable. The COVID-19 pneumonia appears to interfere with the vascular regulation up to complete loss of vascular tone to vasoconstricting or vasodilating agents. We still do not have enough evidence to understand when and on which patients it should be applied. Nitric oxide should not work in fully vasoplegic patients (type 1 in our model) but possibly works in patients in which pulmonary hypertension is more likely (type 2 in our model).

##### (Micro)thrombosis and D-dimer levels

In this disease, thrombosis and associated ischemic events are very common. A daily check of coagulation parameters, in particular D-dimer levels, should be performed in both the type 1 and the type 2 patients, judiciously anticoagulated when indicated.

Type 1 patients:

- PEEP levels should be kept lower in patients with high pulmonary compliance
- Tidal volume thresholds should not be limited at 6 ml/kg
- Respiratory rate should not exceed 20 breaths/min
- Patients should be left “quiet”; avoiding doing too much is of higher benefit than intervening at any cost.

Type 2 patients:

- Standard treatment for severe ARDS should be applied (lower tidal volume, prone positioning, and relatively high PEEP).

## Data Availability

Not applicable.
